# The Misunderstood Coagulopathy of Liver Disease: A Review for the Acute Setting

**DOI:** 10.5811/westjem.2018.7.37893

**Published:** 2018-08-08

**Authors:** Michael F. Harrison

**Affiliations:** Henry Ford Hospital, Department of Emergency Medicine, Department of Internal Medicine, Department of Critical Care Medicine, Detroit, Michigan

## Abstract

The international normalized ratio (INR) represents a clinical tool to assess the effectiveness of vitamin-K antagonist therapy. However, it is often used in the acute setting to assess the degree of coagulopathy in patients with hepatic cirrhosis or acute liver failure. This often influences therapeutic decisions about invasive procedures or the need for potentially harmful and unnecessary transfusions of blood product. This may not represent a best-practice or evidence-based approach to patient care. The author performed a review of the literature related to the utility of INR in cirrhotic patients using several scientific search engines. Despite the commonly accepted dogma that an elevated INR in a cirrhotic patient corresponds with an increased hemorrhagic risk during the performance of invasive procedures, the literature does not support this belief. Furthermore, the need for blood-product transfusion prior to an invasive intervention is not supported by the literature, as this practice increases the risk of complications associated with a patient’s hospital course. Many publications ranging from case studies to meta-analyses refute this evidence and provide examples of thrombotic events despite elevated INR values. Alternative methods, such as thromboelastogram, represent alternate means of assessing in vivo risk of hemorrhage in patients with acute or chronic liver disease in real-time in the acute setting.

## INTRODUCTION

Liver disease presents a major burden on healthcare systems in both North America^1^,^2^ and Europe^3^ and can result in more than 70,000 annual visits to the emergency department (ED).^4^ Liver disease in the setting of acute liver failure (ALF)^5^ or trauma in a patient with cirrhosis^6^–^8^ are predictors of increased mortality and poor patient outcome. One of the challenges these patients pose to healthcare providers in acute settings, such as sepsis and trauma, relates to the coagulopathy of liver disease – specifically, is an individual patient at an increased risk of a spontaneous hemorrhagic event or hemorrhagic procedural complication? The commonly accepted paradigm - increased risk of hemorrhagic events in the setting of elevated international normalized ratio (INR) - is being challenged though it still widely influences day-to-day practice.^9^,^10^

The most commonly used tests for identifying and monitoring coagulopathy include partial thromboplastin time (PTT), prothrombin time (PT), and INR. INR, a ratio of the patient’s PT as compared to a laboratory normative PT value, was designed as a method of monitoring individual patient responses to anticoagulation therapy with a vitamin-K antagonist such as warfarin.^11^ Despite this, tests including INR are often incorrectly applied clinically as a general indication of a patient’s overall bleeding risk due to the ease with which the results are obtained and interpreted. This is particularly true in patients with chronic liver disease and cirrhosis.^12^ However, the utility of INR with respect to predicting risk of hemorrhagic event in chronic liver patients has been refuted^13^–^15^ and warrants further review. An early study concluded that isolated evaluation of bleeding or clotting time is of little prognostic value in patients with liver disease during pre-operative screening.^16^ Given that this study is nearly a half-century old, why are many clinicians still making important clinical decisions based on the interpretation of an INR value in patients who are not being anticoagulated with a vitamin-K antagonist? More specifically, how did the medical community arrive at the commonly accepted “INR less than 1.5” as a safe threshold for invasive procedures?

The liver is responsible for the synthesis of many of the procoagulant and anticoagulant proteins responsible for maintaining hemostasis.^17^ Liver dysfunction is often assumed to be associated with increased bleeding risk, but evidence suggests that other factors such as sepsis, hepatorenal syndrome, hypotension, and endothelial dysfunction contribute to this bleeding tendency rather than isolated cirrhosis and liver disease.^10^,^18^ In most cases, a “rebalancing” occurs and the vast majority of chronic liver disease patients achieve a hemostatic equilibrium.^10^,^15^,^19^–^21^ In cases of traumatic injury or prior to surgical procedures, the measured coagulopathy as assessed by INR is often reversed with pharmaceutical agents (e.g., vitamin K, prothrombin complex concentrate) or transfused blood products (e.g., plasma or platelets). However, this practice of prophylactic transfusion to minimize the risk of hemorrhagic complications is not evidence based despite its wide acceptance.^15^,^19^,^21^

Prophylactic transfusions may expose the patient to increased risk of adverse events (e.g., transfusion reactions including transfusion-related acute lung injury [TRALI] and exacerbation of portal hypertension) as a result of the transfusion, while providing no protective effects.^19^,^22^,^23^ PT and INR analyses assess isolated clotting pathways in vitro despite our knowledge that in vivo clotting pathways do not function in isolation.^24^ As a result, significantly different INR results can be obtained from the analysis of a sample of blood from a cirrhotic patient based on the commercially available thromboplastin used in performing the analysis.^25^ This review intends to address these issues as they pertain to practice in the acute setting such as an ED, a trauma surgeon’s operating room, or an intensive care unit (ICU).

## METHODS

The author conducted a comprehensive search of the relevant literature as it related to chronic liver disease, cirrhosis, ALF, and hemostasis. Searches were performed using PubMed, OVID, Web of Science, Google Scholar, and the Cochrane Library databases. The following criteria were used to search these databases:

1. Access to full-text articles, reports, books, and book chapters in English.
2. Inclusion of a combination of at least two of the terms “coagulopathy,” “INR,” “cirrhosis,” “chronic liver disease,” “acute liver failure.” A secondary search was performed using at least two of the terms listed previously in combination with at least one of the following: “hemorrhage,” “bleeding,” “emergency department,” “trauma,” “central venous catheter,” “lumbar puncture,” “thoracentesis,” “paracentesis,” “procedure,” and “surgery.”

The bibliography of each publication was reviewed to identify any relevant sources that were not identified using the primary search strategies indicated. The author identified over 5,000 articles with these search criteria; many of these were duplicates between search engines and many more related specifically to the perioperative period and management of liver transplantation. A total of 89 articles were reviewed in the final manuscript preparation; these included 76 full-text articles and textbook chapters specific to the search terms above and 13 articles related to the clotting cascade, rates of morbidity and mortality in patients without liver disease and its associated coagulopathy, and statistics specific to the prevalence of and morbidity and mortality of liver disease. In total, the author included in the final manuscript preparation 71 references that were most applicable to the aim of the paper (i.e., the acute setting specific to patients in the ED or the ICU with coagulopathy due to liver disease) and published in full-text English.

## RESULTS

### Pathophysiology of Coagulopathy in Liver Disease

The liver is responsible for the synthesis of nearly all clotting factors and their inhibitors^9^,^12^,^17^ (Table). As a result, patients with chronic liver disease and cirrhosis experience a rebalancing of their hemostatic variables.^15^ Patients in ALF likely experience minimal effects on their in vivo coagulation profiles as assessed with thromboelastography (TEG) despite mean INR values >3.^26^ Furthermore, these patients have significant rates of hypercoagulable (35%) and hypocoagulable (20%) states.^12^ To further complicate matters, the presence of a hypercoagulable state does not exclude the presence of a tendency toward increased bleeding risk, and conversely, increased bleeding risk does not rule out the development of a new thrombus.^27^,^28^ Publications have discussed exactly this paradoxical phenomenon.^28^,^29^

Overall, compensated and decompensated cirrhotic, non-septic patients live in either a balanced homeostatic state or, due to the systemic inflammation associated with liver dysfunction, a prothrombotic state.^10^,^12^,^17^,^20^,^24^,^30^ This concept has been demonstrated and validated using TEG.^26^,^30^ Clinically this phenomenon is often demonstrated by the prevalence of portal vein thromboses^31^ and increased frequency of catheter clotting events during renal replacement therapy.^12^ More specifically, serum levels of antithrombin, protein C, and protein S range from 30–65% of normal; this is comparable to levels observed in patients with inherited deficiencies.^17^ In addition to decreased production of pro- and anticoagulant factors, cirrhotic patients often live in a chronic consumptive state that further decreases these already-low levels of factors on both sides of the clotting spectrum.^27^ In summary the risk of thrombotic events thus may exceed the risk of hemorrhage, and prophylactic anticoagulant therapy – currently regarded as contraindicated in liver disease – may actually provide therapeutic benefit.^10^

### Risk of Hemorrhagic Events with Procedures, Trauma, and Critical Illness

The primary concern related to the elevated INR often observed in cirrhotic patients relates to either unintended or uncontrollable bleeding despite literature suggesting this to be a rare event.^32^ While the INR is often the variable that surgical and interventional services will cite while expressing their concerns about procedural safety,^33^,^34^ platelet concentration and platelet function is a more concerning factor in influencing bleeding risk in this population.^13^,^17^ Regardless, in practice elevated INR is often considered a contraindication for procedural intervention including liver biopsy, intracranial pressure monitor placement, central venous catheter (CVC) placement, paracentesis, thoracentesis, and lumbar puncture.^11^,^17^

The guidelines in both the anesthesiology and the interventional radiology literature, based on a Delphi consensus panel, recommend transfusions in patients with liver disease to correct coagulopathy as determined by INR measurement. The initial guidelines recommended transfusion to correct to an INR≤1.5,^33^,^35^ but more recent guidelines were updated to recommend transfusions to achieve a goal of INR≤1.5 for moderate to significant bleeding risk procedures and INR≤2.0 for low risk procedures.^34^ However, these practices are not supported as evidence based.^15^,^19^ Nonetheless, these recommendations persist despite knowledge that INR results may differ by as much as 0.7 depending on the assay, based on a study of 150 patients, seven commercially available reagents, and four different calibrator sets.^34^,^36^ Intrasubject results for INR values demonstrated statistically significant differences (p<0.001) for 17 of the 21 possible permutations (reagent x calibrator).^36^

In a large prospective study (N=658) of critically ill cirrhotic patients with elevated INR (peak = 17) and thrombocytopenia (nadir = 9 x 10^9^/L), who required CVC placement for the purposes of intravenous access, fluid resuscitation, or initiation of temporary dialysis,^13^ the single major complication in the placement of CVC placement without the assistance of ultrasound guidance in either the subclavian or the internal jugular vein was secondary to the unintended puncture of the subclavian artery. Patient safety in the setting of cirrhotic coagulopathy during invasive procedures can be further augmented with the use of guidance from ultrasound or other imaging modalities.^22^,^23^,^37^ Overall, there is little strong evidence to support the predictive value of abnormal coagulation test results with respect to bleeding with invasive procedures.^14^

Reviews of studies of procedures such as bronchoscopy, femoral angiography, liver biopsy, renal biopsy, thoracentesis, lumbar puncture, and dental extraction also do not support the concept that elevated INR due to liver disease is associated with increased risk of hemorrhagic events.^14^,^38^,^39^ Overall, the risk of hemorrhage in minor procedures that can be performed at bedside is <3% with <1% risk of major bleeding events; in those rare cases of major hemorrhagic complication, mortality may be as low as 0.016%.^17^,^32^,^39^,^40^ To further discredit the utility of INR in predicting these events, it has been reported that the majority of these events, especially in percutaneous liver biopsy procedures, occur in patients with what would be accepted as a normal INR value (INR<1.3).^14^,^24^,^38^

The overall mortality risk in this population, however, is substantial, and one study goes so far as to recommend the consideration of ICU admission for all cirrhotic patients being admitted to the hospital.^6^ Cirrhotic patients with blunt abdominal trauma are significantly more likely to experience injuries that require operative management and experience post-operative complications associated with significant morbidity and mortality.^8^,^41^ Up to a six-fold increase in mortality that approaches 43%, even from minor trauma, has been reported in cirrhotic patients as compared to non-cirrhotic controls.^7^,^8^,^41^,^42^

Predictable tools for risk stratification in liver disease such as Child-Pugh classification and Model for End-stage Liver Disease (MELD) scores correlate well with the increased risk of mortality as a result of trauma^6^,^8^ while trauma-related Injury Severity Scores have been described as grossly inadequate for accurately risk stratifying the cirrhotic trauma patient.^41^ These findings were not necessarily associated with hemorrhagic events, and the occurrence of disseminated intravascular coagulation trended towards significantly increased in cirrhotic patients as compared to controls.^42^ In fact, the serious complications noted often include acute respiratory distress syndrome, pneumonia, renal failure, or sepsis rather than massive hemorrhage.^41^,^42^

### Risk of Thrombotic Events in Critically Ill Patients with Hepatic Dysfunction

A paradox is commonly observed during the care of patients with liver cirrhosis: Despite elevated INR values, clinicians often evaluate for (and subsequently diagnose) portal vein thromboses while clotting of extra corporeal circuits (e.g., hemodialysis or extracorporeal mechanical oxygenation [ECMO]) is a common occurrence in cirrhotic patients.^24^,^27^ Despite the notion of “auto-anticoagulation,” patients with hepatic dysfunction are not protected against the occurrence of venous thromboembolism or other thrombotic events merely by the presence of an elevated PT and INR.^17^,^43^ The increased thrombotic risk in cirrhotic patients is likely attributable to the maintained or even increased capacity for thrombin generation^44^,^45^ or elevations in fibrinogen, FVIII, and von Willebrand factor.^17^ The result is an incidence of 6.3% in one study despite the inclusion of cirrhotic patients with INR>3^43^ and a >50% risk of thrombotic events being identified on autopsy.^17^ In fact, the greatest risk of thromboembolic events was observed in the patients with Child-Pugh Stage C (8.0%).^43^

The risk factors for thrombosis are consistent with elements of Virchow’s triad including procoagulant state, endothelial damage, and turbulent flow; a chronic inflammatory state such as cirrhosis further increases the risk of thrombotic events.^24^,^27^ The procoagulant state is often due to a localized phenomenon of persistently present procoagulant factors due to disrupted hemodynamics^20^ or a decreased hepatic ability to clear activated procoagulant factors.^31^ Given the intricate interplay between factors, platelets, and other physiological conditions, in vitro models to accurately predict in vivo thrombotic events are often inadequate.^20^

### Alternatives for Laboratory Evaluation of Coagulopathy

The elevated PT and INR observed in cirrhotic patients often occurs with a normal or near-normal activated PTT; this is representative of an isolated factor VII or concurrent factor VII / VIII elevations.^17^ The isolated evaluation of PT and INR does not take other defects such as thrombocytopenia and platelet function defects into account,^17^ despite the prevalence and importance of these factors in evaluating for the presence of in vivo coagulopathy in a cirrhotic patient.^9^ Another century-old test of coagulopathy is bleeding time, although the evidence is equivocal regarding is reliability and reproducibility^34^,^46^,^47^ and it is seldom used in modern medicine due to its unreliable utility on the individual patient basis.^47^ However, the proposed benefit of assessing bleeding time is the inclusion of the entire in vivo clotting cascade rather than the incomplete, in vitro coagulation cascade commonly assessed with PT, PTT, and INR evaluation. In ALF patients, PT results and INR calculation do not correlate well with more advanced and specific assessments of coagulation state from tools such as TEG.^12^

TEG represents an alternative to bleeding time, PT measurement, and INR calculation in patients with hepatic dysfunction for whom a provider wishes to evaluate a true coagulation profile that correlates well with the in vivo clinical presentation.^12^,^26^,^31^,^48^ While not yet a “gold standard” technique, it does demonstrate benefit in guiding transfusion-based decisions in elective cardiac procedures^49^ and liver transplantation.^50^ It also provides promising results in the management of acute coagulopathy in critical acute settings such as trauma in the ED,^49^,^51^,^52^ military theater of operations,^53^ and ECMO,^54^ although more research is needed in these settings.

In currently available studies in acute clinical settings,^48^,^54^,^55^ TEG provides a rapid bedside tool to assess and monitor hemostatic characteristics using whole blood samples (Figure). A small amount of whole blood, <5mL, at body temperature (37^O^C) is placed in an oscillating cup after sampling from venipuncture. A pin suspended from a torsion wire couples with the blood as fibrin strands form, and the result is increased wire tension as detected by an electromagnetic transducer. The resulting electrical signal is converted to the TEG trace, which can be displayed in real time on a computer monitor.^30^,^56^ Complete results are available in less than 30 minutes, though preliminary results are available much sooner (<15 minutes).^30^,^56^ This provides the clinician the ability to consider the multiple factors associated with a true coagulopathy including activation of the coagulation cascade, the inhibition of the clotting cascade, fibrinolytic activity, and platelet function.^26^,^48^ This information from a point-of-care tool can guide the transfusion of specific blood products (e.g., platelets, fresh frozen plasma [FFP], cryoprecipitate) or medications (e.g., tranexamic acid)^55^ while minimizing unnecessary medications or blood product transfusion^49^,^54^,^56^,^57^ or predicting mortality^51^ and thrombotic risk^58^ following admission through the ED as a trauma activation.

Stravitz^26^ provides an excellent summary with examples of TEG curves during a variety of clinical scenarios (thrombocytopenia, acute hepatic failure, decompensated cirrhosis, etc.) while da Luz et al.^55^ provide similar information in the context of a trauma patient. The correlation of TEG results with dynamic risk of bleeding has been demonstrated during the course of a patient’s hospitalization.^31^ A pitfall of TEG must be recognized: given the dynamic state in which a cirrhotic patient and their coagulation profile exist, a baseline TEG result obviously does not accurately predict bleeding or thrombotic risk over a follow-up period measured in months or years.^30^ It would not be unreasonable to assume that a critically ill, hospitalized patient with cirrhosis would require repeated TEG assessments during the course of their resuscitation and treatment. The utilization of TEG is associated with an increased cost as compared to ordering a laboratory test such as PTT,^54^ though this cost may be in the order of $22 United States dollars per test.^39^ Overall, TEG does provide trends toward improved hemostasis, decreased anticoagulant or blood product requirements, and improved patient outcomes^39^,^54^ through which these additional costs may be quickly recouped. As a result, TEG has been described as cost-effective overall.^56^

Specific to liver disease, TEG-guided transfusion protocols during liver transplantation decrease the amount of bleeding but have no effect on overall mortality.^50^ Similarly, TEG can predict post-operative thrombus risk in these patients.^50^ With respect to acute procedural setting such as central line placement, a small nonrandomized prospective study (N=90) demonstrated TEG’s ability to predict bleeding (n=11) in patients with cirrhosis and abnormal INR results during blind central line placement.^59^ Additionally, the INR cut off for bleeding risk in this same study was 2.6. Overall, the majority of the TEG studies and specifically those specific to liver disease are small and not without limitations. Obviously prospective, randomized studies would strengthen the case for TEG’s utility, given the plethora of literature that indicates the lack of utility of traditional laboratory studies of coagulation. The potential benefit of TEG with respect to point-of-care assessment of whole blood coagulation characteristics makes it a tool worthy of further study with larger populations in randomized controlled studies.

### Management Options for Coagulopathy

A small study in a broad population of ED, surgical, general medical ward, and ICU patients demonstrated that the use of FFP to correct mild elevations in PT and INR only corrected the values to baseline in 0.8% of patients, while only 15.9% of this population achieved a 50% correction in PT and INR values.^60^ These results are consistent with findings presented in multiple review papers on the topic^17^,^32^ with one authoritative source bluntly stating that the transfusion of these products only provides partial and transient correction but never a complete correction of the laboratory derangements regardless of the number of FFP units transfused.^19^ The transient mean change in INR as a result of transfusion ranges from 0.03 to 1.3 per unit of FFP,^24^ and the effect is described as “trivial” because the transfusion of FFP “fails to correct the PT in 99% of patients.”^60^ Low-dose recombinant factor VIIa therapy has been associated with improved outcomes and decreased transfusion requirements in trauma patients with coagulopathy.^61^

It would appear the best management of suspected coagulopathy, as assessed by INR and whether the patient is actually hyper- or hypocoagulable, is the treatment of the underlying cause for the hepatic and synthetic dysfunction.^5^ Given the limited utility of INR as a tool of assessing synthetic function in a cirrhotic patient, this might include administering vitamin K in an effort to augment synthetic function of clotting fators.^17^,^32^ However, the clinical benefit of this approach may not be predictable as the absorption of vitamin K (and A, D, and E) is dependent on bile production,^24^ a process that is complex in itself but generally accepted to be decreased in the setting of cirrhosis.^31^,^62^,^63^ On a more positive note, multiple studies have demonstrated that a surprisingly small proportion, generally <15%, of cirrhotic patients are truly vitamin-K deficient.^24^ This provides further evidence that INR, a tool designed to monitor vitamin-K antagonism, is inappropriate for assessing the coagulopathy of cirrhotic patients.

The safety threshold of achieving and maintaining an INR<1.5 in patients prior to non-emergent invasive procedures was derived from a report by the American Society of Anesthesiologists Task Force on Blood Component Therapy.^35^ A review by Ng^24^ describes this “incorrectly” derived and accepted target value while chronicling subsequent publications demonstrating insufficient evidence to support prophylactic blood product transfusions to optimize INR. A major risk of blood product transfusions to correct an elevated INR in the setting of hepatic dysfunction is due to the lack of efficacy and inability to accurately assess the transfusion-related risk borne by the patient. While the risk associated with transfusion-associated reactions such as TRALI or hemolysis is significantly lower than the 1–3% risk of hemorrhage in minor procedures that can be performed at bedside, it should be noted that many transfusion-associated events are under-reported and the benefit, as summarized in the prior section to be often transient or minimal, does not outweigh the risk.^17^,^32^,^60^,^64^–^66^

In patients with liver disease in particular, the prophylactic transfusion of cryoprecipitate has been associated with an increased risk of thrombotic events in end-stage liver disease (ESLD) patients^17^ and thus should be avoided if not absolutely necessary. Administering factor VIIa may be considered if FFP and vitamin K has not corrected the coagulopathy, but care should be taken to avoid treating simply to correct an abnormal laboratory result.^17^,^32^ Other recombinant techniques such as plasma exchange have only demonstrated utility in pre-operative settings in preparation for liver transplantation.^17^ The evidence published since the American Society of Anesthesiologists Task Force on Blood Component Therapy^35^ recommendation of maintaining an INR <1.5 now suggests, as reviewed and summarized in Ng,^24^ that procedural safety is achievable with INR values ranging from 2.5 to 4.0.

The final aspect of the management of coagulopathy in cirrhotic patients with elevated INR values is prophylactic anticoagulation for venous thromboembolism (VTE). Hospitalized patients with liver disease develop a deep vein thrombosis or pulmonary embolism (PE) at rates of 4–12% despite standard-of-care prophylaxis;^27^ hospitalized cirrhotic and noncirrhotic liver disease patients may experience new VTE at a rate of up to 6% regardless of INR.^43^,^67^ The risk of VTE is greater than the risk of PE, although the etiology of this discrepancy is not well understood.^10^,^67^,^68^ The relative risk for VTE in cirrhotic patients is reported to be >2^68^ and associated with greater mortality in higher Child-Pugh stages.^43^ The best predictor of VTE in a cirrhotic patient assumed to be “auto-anticoagulated” based on an elevated INR value is serum albumin; it is hypothesized that lower serum albumin concentration is a surrogate for decreased protein synthesis by the liver and thus decreased production of endogenous anti-coagulant factors such as Protein C and S.^67^ This is concerning as some studies report rates of prophylactic anticoagulation in this population to be as low as only 21%.^27^,^43^

Unfortunately, the available literature focuses on the under-recognized need for anticoagulation and the current misconception related to “auto-anticoagulation.” The guidelines, however, do not provide the needed specifics related to the prophylactic approach in complex clinical scenarios such as caring for critically ill patients with cirrhosis.^50^,^69^,^70^ Perhaps recognizing the misconception will be the first step toward the research and attention required to create guidelines related to these specific patients and scenarios.

## DISCUSSION

Hemostasis in cirrhotic patients is a dynamic balance.^15^,^24^ In the majority of clinical scenarios, patients with cirrhosis and impaired protein synthesis achieve hemostasis despite elevated INR values^20^ and may be more prone to thrombotic or thromboembolic events.^27^,^43^,^67^ The best application of INR to a patient with liver disease is to monitor the degree of impairment of synthetic function^12^ or to predict mortality.^43^ Predictive scores such as MELD make use of INR for this specific purpose in ESLD,^71^ though this may have specific challenges based on the variation in results dependent upon the commercially available thromboplastin used in the analysis;^25^ the universality of the results may not be as robust as widely assumed.

The commonly accepted dogma in the ED that an elevated INR is associated with increased risk of hemorrhagic events while protected from thrombotic complications is not supported by the literature^10^,^15^ or by the underlying theory of INR testing. Furthermore, guidelines such as “INR≤1.5” are merely expert opinion that are not supported by more recent, evidence-based publications and may expose patients to more risk if prophylactic blood product transfusions occur in the futile pursuit of a transient decrease in INR.^24^ Unlike other coagulopathies observed in ED and ICU settings such as hemophilia where life-threatening bleeding is a real and serious concern, cirrhotic patients often have rebalanced hemostasis and do not hemorrhage at the rates many clinicians wrongly assume to be the case.^10^,^15^,^24^,^57^ The recognition of this commonly accepted pitfall will be the first step to addressing a number of questions: what is the best method by which to accurately assess the coagulopathy associated with liver disease?; and what is the threshold at which the risk/benefit ratio is exceeded for a specific procedure such as central line or lumbar puncture?

## LIMITATIONS

Medicine’s understanding of the physiology associated with normal coagulation stems from studies of rare congenital clotting disorders such as hemophilia A or factor VIII deficiency.^21^ Studies with patients in these populations have not been able to identify thresholds of safe limits for individual clotting factor deficiencies, though the commonly accepted limit is to maintain clotting factor deficiencies at a level of >1%.^24^ Given the deficiency in multiple coagulation factors in a cirrhotic presentation, vitamin K-dependent clotting factor deficiency (VKCFD) is thought to be a superior, naturally occurring analogue to hemophilia in assessing the bleeding risk associated with surgical procedures or trauma in the setting of an elevated PT or INR.^24^ However, this analogue is not perfect and the natural history of VKCFD “suggests factors other than simple clotting-factor deficiencies alone predispose to bleeding.”^24^ When the multiple factors involved in thrombotic and thrombolytic events are considered as in the Table, the complexity of predicting “who will bleed” and “who will clot” becomes evident; it becomes even more evident that, as reported by Donaldson et al.,^16^ a simple test of only one pathway is inadequate to accurately make this prediction.

## CONCLUSION

In patients with abnormal coagulation testing results in the setting of liver disease, INR and PT may be best used to provide the practitioner with information about the synthetic function of the liver but not to assess hemorrhagic risk. The evidence supports a “watchful waiting” approach to the transfusion of platelets and fresh-frozen plasma with a bedside assessment of the patient’s actual hemorrhagic risk. The safest assumption that a practitioner in an acute and critical setting can make about any cirrhotic patient is that, even on their healthiest day, they are at an elevated risk of adverse outcomes that may be associated with an adverse thrombotic rather than the commonly feared catastrophic hemorrhagic event.

## Figures and Tables

**Figure 1:**
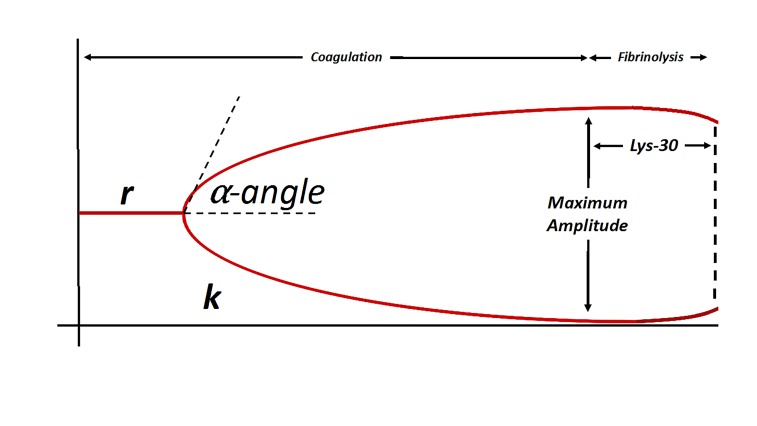
Example of thromboelastogram analysis curve (adapted from Stravitz et al, 2012^26^). r – measured in minutes, the reaction time (r) represents the latency period between the initiation of the reaction and the initiation of fibrin formation, represented by *k; k* – measured in minutes, the kinetic time (k) represents the time required to reach a clot strength of 2mm; *α*-angle – measured in degrees, corresponds to kinetics of clot formation; a steeper angle corresponds to a more rapid rate of clot formation. Maximum amplitude – measured in mm, represents the maximum clot strength and is a function of both platelet count / function and fibrinogen concentration; Lys-30 – represents the rate of clot degradation in the 30-minute period following the achievement of maximum clot strength as represented by maximum amplitude.

**Table:** Summary of factors associated with hemostasis (compiled^9^,^10^,^24^,^31^).

Procoagulants		Anticoagulants		Fibrinolytics	
		
Hepatic synthesis	Non-hepatic synthesis	Hepatic synthesis	Non-hepatic synthesis	Hepatic synthesis	Non-hepatic synthesis
Factors:	Factors:	Proteins:	Tissue factor pathway inhibitor	Plasminogen (zymogen) and plasmin	
I	VIII*	C			
II(prothrombin)	von Willebrand (vWf)	S			
III		Z			
IV					
V	Platelets**				
VI		Anti-thrombin III			
VII	Anti-phospholipid antibodies***				
VIII*					
IX					
X					
XI					
XII					
Fibrinogen					

* Factor VIII is synthesized primarily by hepatic sinusoidal endothelial cells, but a sizeable proportion of the synthetic process also occurs in non-hepatic sinusoidal cells. As a result, liver disease does not decrease plasma concentrations of von Willebrand factor (vWf); the chronic inflammation associated with chronic liver disease may actually increase plasma concentrations of vWf.^10^,^31^

** Decreased in circulating number and function in liver disease.

*** Increased in liver disease.
